# Exploring the plasmatic platelet-activating factor acetylhydrolase activity in patients with anti-phospholipid antibodies

**DOI:** 10.1007/s13317-017-0092-7

**Published:** 2017-03-25

**Authors:** Martina Fabris, Adriana Cifù, Cinzia Pistis, Massimo Siega-Ducaton, Desrè Ethel Fontana, Roberta Giacomello, Elio Tonutti, Francesco Curcio

**Affiliations:** 1 Department of Medical and Biological Sciences, University of Udine, Padiglione CSL - Via Chiusaforte, Ingresso F3, 33100 Udine, Italy; 2 Institute of Clinical Pathology, Department of Laboratory Medicine, University Hospital of Udine, Padiglione CSL - Via Chiusaforte, Ingresso F3, 33100 Udine, Italy; 3 Laboratory of Immunopathology and Allergy, Department of Laboratory Medicine, University Hospital of Udine, Udine, Italy

**Keywords:** Platelet-activating factor acetylhydrolase, Anti-phospholipid syndrome, Anti-beta2-glycoprotein I antibodies, Anti-prothrombin/phosphatidylserine antibodies, Lupus anticoagulant, Atherosclerosis

## Abstract

**Purpose:**

To explore the role of plasmatic platelet-activating factor acetylhydrolase (PAF-AH), a marker of cardiovascular risk, in patients with anti-phospholipid antibodies (aPL).

**Methods:**

PAF-AH activity was assessed in a series of 167 unselected patients screened for aPL in a context of thrombotic events, risk of thrombosis or obstetric complications and in 77 blood donors.

**Results:**

116/167 patients showed positive results for at least one aPL among IgG/IgM anti-prothrombin/phosphatidylserine (aPS/PT), anti-cardiolipin (aCL), anti-beta2-glycoprotein I (aβ2GPI) or lupus anticoagulant (LAC), while 51/167 patients resulted aPL-negative. LAC+ patients disclosed higher PAF-AH than LAC-negative (22.1 ± 6.4 nmol/min/ml vs. 19.5 ± 4.1 nmol/min/ml; *p* = 0.0032), and aPL-negative patients (*p* = 0.03). Patients presenting positive IgG aβ2GPI disclosed higher PAF-AH than patients with only IgM aβ2GPI-positive antibodies (23.1 ± 7.2 nmol/min/ml vs. 20.1 ± 5.3 nmol/min/ml; *p* = 0.035), as well as than patients showing only isolated LAC, aCL or aPS/PT (16.9 ± 3.8 nmol/min/ml; *p* = 0.003).

**Conclusions:**

PAF-AH plasmatic activity is particularly up-regulated in LAC+ and in aβ2GPI IgG+ patients, possibly representing an alternative prognostic biomarker for the therapeutic management of APS patients.

## Introduction

Anti-phospholipid syndrome (APS) is a hypercoagulable disorder clinically displayed by venous or arterial thrombosis and/or adverse obstetric events, accompanied by persistent and elevated levels of anti-phospholipid antibodies (aPL) [1]. According to the 2006 revised international classification criteria [2], patients with definite diagnosis of APS are those presenting positive lupus anticoagulant (LAC) and/or one among anti-cardiolipin (aCL) IgG or IgM or anti-beta2 glycoprotein I (aβ2GPI) IgG or IgM antibodies. However, during the last international congress on aPL antibodies, the major experts defined the role of other so-called “non criteria” antibodies, contributing to assess the risk of thrombosis or the identification of potential seronegative APS, such as the anti-prothrombin/phosphatidylserine antibodies (aPS/PT) [3]. Of note, the combination of aβ2GPI, aPS/PT and LAC has demonstrated the best diagnostic accuracy for APS [4] and aPS/PT were recently recommended as a surrogate for LAC when specific inhibitors and/or analytical variables may affect its interpretation [5]. However, no definite recommendations are available to guide the therapeutic approach in patients positive only for aPS/PT antibodies.

Platelet-activating factor acetylhydrolase (PAF-AH) is a family of enzymes, the most abundant of which is the plasma form, also called lipoprotein-associated phospholipase A2 (Lp-PLA2) [6]. The plasmatic PAF-AH is constitutively active and circulates bound to LDL, HDL and other lipoproteins and catalyses the hydrolysis of the sn2 acetate of PAF and PAF mimetics, which are early mediators of inflammation [7]. PAF activates a variety of cells of the innate immune system promoting migration, adhesion and inflammatory effects. Thus, PAF-AH while inactivating PAF is considered an important factor in preventing an exaggerated inflammatory response and in protecting cells from uncontrolled oxidative damage [8].

Several studies reported a significant association between higher PAF-AH plasmatic activity and the severity of cardiovascular (CV) disease and identified PAF-AH as a marker of vascular inflammation and atherosclerotic plaque instability [9–11].

More and more papers in recent literature emphasize the relevant link between endothelial dysfunction, atherosclerosis and APS [12–14]. Chronic inflammation is involved in various stages of development of the atherosclerotic plaques. Among the key molecules involved in the atherosclerotic process are heat-shock proteins, oxidized LDL (oxLDL) and β2GPI. The latter is identified as an anti-atherogenic agent involved in the atheromatous plaque formation in APS patients, since it is targeted by the aβ2GPI antibodies, typically associated with APS [15, 16].

In this study we analysed PAF-AH plasmatic activity in a large series of unselected patients screened for aPL antibodies in a reference laboratory for the diagnosis of autoimmune diseases, investigating its association with different pattern of aPL positivity.

## Materials and methods

### Patients

The study was conducted in 167 consecutive unselected patients (124 females and 69 males; mean age: 51 ± 16 years) who were screened for the presence of aPL at the Laboratory of Immunopathology of the University Hospital of Udine in the context of routine testing for thrombotic events, risk of thrombosis or obstetric complications. Patients were compared to 77 blood donors (BDs; 39 females and 38 males; mean age: 39 ± 13 years) enrolled at the Transfusion Unit of the same Hospital. All patients and controls gave their informed consent to the study according to the Declaration of Helsinki (1964) and to the Italian legislation (Authorization of the Privacy Guarantor No. 9, 12th of December 2013).

## Methods

The plasmatic PAF-AH activity was assessed by a colorimetric assay (PAF-AH Assay Kit-Cayman Chemical Company, Ann Arbor, MI, USA). Briefly, plasma or serum samples were incubated with the 2-thio PAF substrate, i.e., hydrolyzed by PAF-AH at the sn2-position releasing free thiols detected by DTNB Ellman’s reagent (5,5′-dithio-*bis*-2-nitrobenzoic acid).

Anti-cardiolipin (aCL) and anti-beta2 glycoprotein I (aβ2GPI) IgG/IgM antibodies were investigated in all patients, while 125 patients were tested for lupus anticoagulant and 125 for anti-phosphatidylserine/prothrombin (aPS/PT) IgG/IgM antibodies. aCL and aβ2GpI antibodies were analysed by chemiluminescence (Zenit RA instrument by A. Menarini Diagnostics, Italy), while aPS/PT were assayed by Quanta Lite aPS/PT IgG/IgM ELISA kit (Inova Diagnostics Inc, San Diego, CA). Plasma samples were tested for the presence of LAC according to the recommended criteria from the ISTH Subcommittee on Lupus Anticoagulant-Phospholipid-dependent antibodies and optimized according to recently published standardization [17, 18]. Total cholesterol, low-density lipoproteins (LDL), high-density lipoproteins (HDL) and triglycerides were analysed by diagnostic methods.

### Statistic analyses

Quantitative variables were expressed as mean ± standard deviation and checked for normality distribution by the Shapiro–Wilk test. Statistical analyses were performed with GraphPad Prism software. To compare biomarker serum levels between patients and controls, either Mann–Whitney or unpaired *t* test was used when appropriate. Correlation analyses were performed using the Pearson’s or the Spearman’s rank correlation coefficient.

## Results

### PAF-AH plasmatic activity in patients and controls: correlation with lipid metabolic markers

PAF-AH plasmatic activity in BDs disclosed a mean value of 15.6 ± 4 nmol/min/ml (range 5.9–28.4). As expected [11], a significant correlation was found between PAF-AH and total cholesterol (*r* = 0.25; *p* = 0.032), a stronger direct correlation with LDL (*r* = 0.46, *p* < 0.0001) and a highly significant inverse correlation with HDL (*r* = −0.45, *p* < 0.0001). No correlation was found with age and sex (15.5 ± 5 nmol/min/ml in females vs. 15.7 ± 3.3 nmol/min/ml in males).

**Fig. 1:**
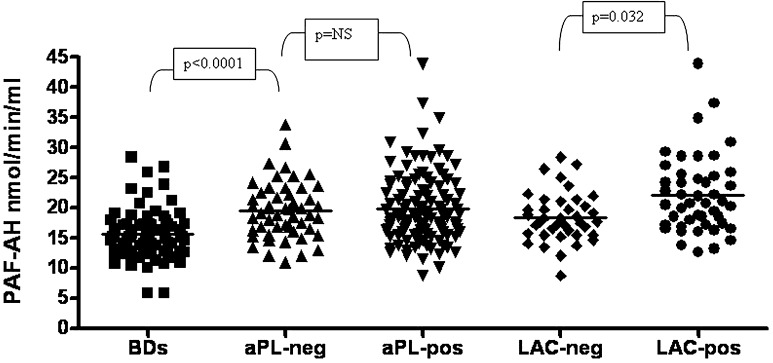
PAF-AH plasmatic activity in patients and controls. PAF-AH plasmatic activity was markedly more elevated in the overall patients (19.8 ± 5.5 nmol/min/ml) than in BDs (*p* < 0.0001), but no difference occurred between aPL-positive and aPL-negative patients (19.9 ± 5.8 nmol/min/ml vs. 19.6 ± 4.7 nmol/min/ml; *p* = ns). LAC-positive patients disclosed higher PAF-AH than LAC-negative (22.1 ± 6.4 nmol/min/ml vs. 19.5 ± 4.1 nmol/min/ml; *p* = 0.0032)

Of the 167 patients undergoing aPL investigation, 116 showed at least one positive aPL among LAC, aCL, aβ2GPI or aPS/PT antibodies, while 51 resulted all negative. PAF-AH plasmatic activity was markedly more elevated in the overall patients (19.8 ± 5.5 nmol/min/ml) than in BDs (*p* < 0.0001), but no difference was found between aPL+ and aPL-negative patients (19.9 ± 5.8 nmol/min/ml vs. 19.6 ± 4.7 nmol/min/ml; Fig. 1).

Of note, total cholesterol levels did not differ significantly between BDs and the overall patients, nor between BDs and aPL+ patients (188 ± 38 mg/dl vs. 198 ± 42 mg/dl; *p* = 0.10) and between aPL+ and aPL-negative patients (206 ± 52 mg/dl; *p* = 0.47). However, LDL serum levels were higher in aPL-negative patients than in BDs (127 ± 42 mg/dl vs. 104 ± 35 mg/dl; *p* = 0.0073) as well as in aPL+ patients (109 ± 35 mg/dl; *p* = 0.032 vs. aPL-negative; *p* = ns vs. BDs).

The significant correlation between PAF-AH activity and cholesterol, LDL and HDL serum levels persisted in aPL+ patients (*r* = 0.21, *p* = 0.041; *r* = 0.23, *p* = 0.024 and *r* = −0.31, *p* = 0.0027, respectively), while in aPL-negative patients it was evident only for LDL (*r* = 0.29, *p* = 0.14; *r* = 0.25, *p* = 0.0027 and *r* = −0.25, *p* = 0.21, respectively).

### PAF-AH plasmatic activity in patients disclosing distinct pattern of aPL positivity

**Fig. 2:**
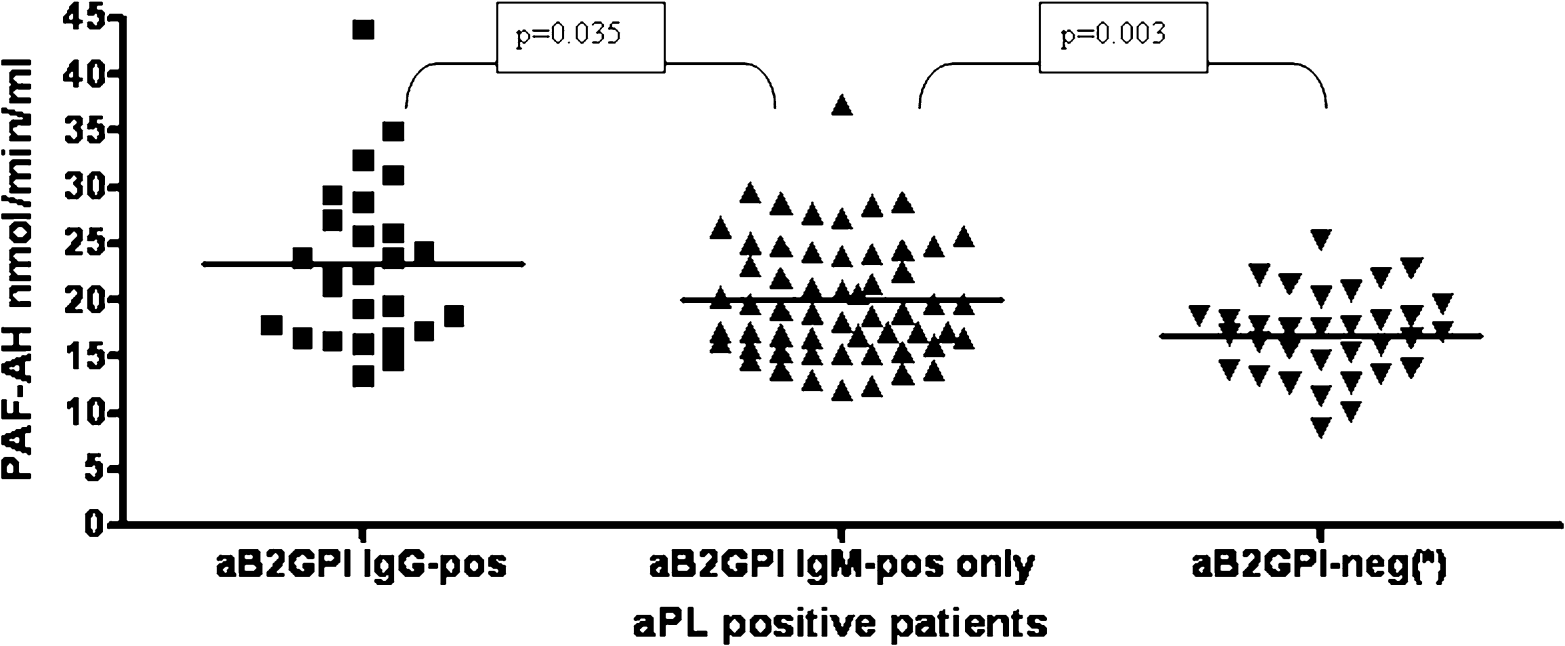
PAF-AH plasmatic activity in patients with distinct aPL positivities. Patients presenting positive aβ2GPI IgG antibodies disclosed higher PAF-AH plasmatic activity than patients presenting only positive aβ2GPI IgM antibodies (23.1 ± 7.2 nmol/min/ml vs. 20.1 ± 5.3 nmol/min/ml; *p* = 0.035). Patients negative for aβ2GPI IgG or IgM antibodies showing either isolated LAC or aCL or aPS/PT-positive antibodies (*) demonstrated significantly lower PAF-AH activity (16.9 ± 3.8 nmol/min/ml; *p* = 0.003 vs. aβ2GPI IgM+)

As shown in Fig. 1, when distinguishing aPL+ patients based on LAC assay, LAC+ disclosed higher PAF-AH than LAC-negative patients (22.1 ± 6.4 nmol/min/ml vs. 19.5 ± 4.1 nmol/min/ml; *p* = 0.0032). Of note, total cholesterol levels did not differ between LAC+ and LAC-negative patients (202 ± 39 mg/dl vs. 201 ± 34 mg/dl; *p* = ns), as well as LDL (113 ± 39 mg/dl vs. 108 ± 26 mg/dl; *p* = ns) and HDL serum levels (60 ± 21 mg/dl vs. 63 ± 21 mg/dl; *p* = ns). Moreover, LAC+ patients disclosed higher PAF-AH than aPL-negative patients (*p* = 0.03), with again no difference with regard to HDL (62 ± 24 mg/dl in aPL-negative; *p* = ns) and LDL (127 ± 42 mg/dl in aPL-negative; *p* = ns). As illustrated in Fig. 2, patients presenting aβ2GPI IgG+ antibodies disclosed higher PAF-AH plasmatic activity than patients presenting only aβ2GPI IgM+ antibodies (23.1 ± 7.2 nmol/min/ml vs. 20.1 ± 5.3 nmol/min/ml; *p* = 0.035), but they did not differ with regard to LDL and HDL serum levels. Patients who were negative for aβ2GPI IgG or IgM antibodies, but who showed either isolated LAC or aCL or aPS/PT-positive antibodies demonstrated significantly lower PAF-AH activities that appeared comparable to those measured in BDs (Fig. 2; 16.9 ± 3.8 nmol/min/ml; *p* = ns vs. BDs; *p* = 0.003 vs. aβ2GPI IgM+). Total cholesterol, LDL and HDL serum levels in patients with isolated LAC or aCL or aPS/PT-positive antibodies did not differ from those measured in patients with aβ2GPI IgM+ or IgG+ antibodies. Overall, aPS/PT IgG+ patients disclosed PAF-AH activity close to that of aPS/PT IgM+ patients (17.3 ± 3 nmol/min/ml vs. 16.1 ± 3.9 nmol/min/ml; *p* = ns). Finally, patients disclosing aβ2GPI IgG+ antibodies together with aPS/PT IgG+ antibodies tended to show higher PAF-AH activity than patients disclosing only aβ2GPI IgG+ antibodies (23.4 ± 7 nmol/min/ml vs. 21 ± 4.7 nmol/min/ml; *p* = ns).

## Discussion

Increased PAF-AH expression demonstrated a predictive role for cardiovascular events in relation to the vulnerability of atherosclerotic plaques. Therefore, PAF-AH dosage has been proposed in the assessment of CV risk, to ensure a better stratification of at risk populations [10]. To date, PAF-AH has never been investigated in the context of APS patients, or, even less, in patients at risk to develop an overt APS (i.e. asymptomatic carriers of aPL antibodies, patients affected by systemic connective tissues diseases).

Our study was originally conducted in a context of patients routinely screened for APS, demonstrating a significant association between the presence of aPL antibodies, LAC and aβ2GPI IgG in particular, and PAF-AH up-regulation in plasma.

Atherosclerosis is definitely recognized as a chronic inflammatory response to the accumulation of lipoproteins in the walls of arteries [19]. PAF-AH, produced by monocytes, macrophages and T lymphocytes, and mainly associated with LDL, is predominantly expressed in the necrotic centre of the atherosclerotic plaques and in the macrophage-rich areas and releases pro-inflammatory mediators, such as lysophospholipids and oxidized fatty acids [8].

Besides the presence of LAC, several different targets of aPL could be determined by a number of analytical methods, with frequent discordant results, that could make the laboratory diagnosis of APS extremely complicated. The main role of the aβ2GPI antibodies, especially those specifically targeting domain I [20], is widely accepted and present results seem to further confirm their importance with regard to CV risk stratification, since PAF-AH appeared particularly elevated in aβ2GPI-positive patients and more so in those displaying LAC activity and carrying the IgG isotype. This particular association may be explained by the fact that IgG aβ2GPI antibodies are able to recognize the stable complex between oxLDL and β2GPI, thus facilitating macrophage-derived foam cell formation in patients with APS [15]. The immune-pathological mechanisms sustained by oxLDL/β2GPI complexes are not yet fully understood, but Toll-like receptor 4 (TLR4) was recently shown to be involved [15]. TLR4 could be the key player linking PAF-AH up-regulation to aβ2GPI IgG antibodies in APS, as evidenced by a mouse model of preterm delivery which demonstrated that PAF effects and signalling depend upon TLR4 stimulation [21].

Lp-PLA2 activity proved to be markedly reduced in vivo when the enzyme is bound to HDL [8], and this is in line with our observation that aβ2GPI IgG+ patients disclosed higher PAF-AH and lesser HDL than BDs. This is not true for other subgroups of patients, such as aPL-negative patients or those presenting only isolated LAC or aCL or aPS/PT antibodies. Compared to these patients, PAF-AH plasmatic activity up-regulation in aβ2GPI IgG+ cases appeared to be at least partially disconnected from the lipoprotein levels and specifically linked to the presence of such aPL antibodies.

Therefore, PAF-AH up-regulation arose as a specific thrombotic risk marker in patients carrying aβ2GPI antibodies and is not generally associated with other aPL antibodies possibly implicated in APS manifestations, but further studies are needed to confirm this observation.

Unfortunately, in two large randomized clinical trials, an inhibitor of PAF-AH (darapladib) [22, 23] failed to reduce the risk of major coronary events as compared to placebo. In addition, it was associated with significantly higher rates of drug discontinuation and adverse effects. These results suggested that PAF-AH may be a biomarker of vascular inflammation, rather than a causal pathway of CV diseases [23]. Therefore, high PAF-AH activity could reflect a response to pro-inflammatory stress characteristic both of atherosclerosis and APS [24].

The leading cause of death in primary and secondary APS patients are cardiovascular events due to accelerated atherosclerosis, which often progresses more rapidly, compared with the general population [14]. Some key pro-inflammatory proteins correlate with APS clinical manifestations [25] and common radiological markers of subclinical atherosclerosis and CV risk were often reported in such patients [13]. However, to date, besides the presence of aPL itself, no serological biomarkers specifically associated with aPL-related pathogenic mechanisms have been identified as useful to improve the classification of CV risk in aPL+ patients, with and without overt clinical manifestations.

In this scenario, present findings on PAF-AH assume a relevant place, possibly representing a reliable and affordable biomarker useful to identify patients at higher risk in which to take a more cautious therapeutic attitude in the follow-up.

Moreover, studying PAF-AH metabolic pathway may help to better explain the pathogenesis of APS and to improve management and interpretation of aPL-related issues, from the analytical results, to the final therapeutic decision.

We and others recently demonstrated an important role of aPS/PT antibodies in the serological diagnosis of APS [5, 26]; however, the therapeutic management of patients characterized by the presence of isolated aPS/PT remains an open issue. Patients with isolated aPS/PT antibodies disclosed lower, BDs-like, PAF-AH as compared to patients with positive aβ2GPI antibodies. Nevertheless, aPS/PT antibodies may exert their distinct pathogenic role through pathways in which PAF-AH is not involved. Anyhow, in case aPS/PT IgG (but not IgM) are combined to aβ2GPI IgG antibodies, PAF-AH tends to be further up-regulated.

In conclusion, the prognostic information conveyed by plasmatic PAF-AH activity in patients with positive aPL antibodies appeared to be independent of common lipid metabolic markers (i.e. LDL), as previously reported by other authors in the context of patients with major coronary events [11]. The major international scientific societies of cardiologists have included the measurement of PAF-AH activity among the biomarkers useful in risk stratification of adult asymptomatic patients at intermediate cardiovascular risk in their guidelines [27].

Our data are encouraging, but since some cohort comparison include partially overlapping data, a definite utility of PAF-AH plasmatic activity as a new prognostic biomarker also in patients with aPL antibodies and/or definite APS is actually precluded. For this reason further prospective studies on selected patients are ongoing.

## References

[1] Khamashta M, Taraborelli M, Sciascia S, Tincani A (2016). Antiphospholipid syndrome. Best Pract Res Clin Rheumatol.

[2] Miyakis S, Lockshin MD, Atsumi T, Branch DW, Brey RL, Cervera R, Derksen RH, de Groot PG, Koike T, Meroni PL, Reber G, Shoenfeld Y, Tincani A, Vlachoyiannopoulos PG, Krilis SA (2006). International consensus statement on an update of the classification criteria for definite APS syndrome. J Thromb Haemost.

[3] Bertolaccini ML, Amengual O, Andreoli L, Atsumi T, Chighizola CB, Forastiero R, deGroot P, Lakos G, Lambert M, Meroni P, Ortel TL, Petri M, Rahman A, Roubey R, Sciascia S, Snyder M, Tebo AE, Tincani A, Willis R (2014). 14th International Congress on Antiphospholipid Antibodies Task Force. Report on antiphospholipid syndrome laboratory diagnostics and trends. Autoimmun Rev.

[4] Sciascia S, Murru V, Sanna G, Roccatello D, Khamashta MA, Bertolaccini ML (2012). Clinical accuracy for diagnosis of antiphospholipid syndrome in systemic lupus erythematosus: evaluation of 23 possible combinations of antiphospholipid antibody specificities. J Thromb Haemost.

[5] Fabris M, Giacomello R, Poz A, Pantarotto L, Tanzi N, Curcio F, Tonutti E (2014). The introduction of anti-phosphatidylserine/prothrombin autoantibodies in the laboratory diagnostic process of anti-phospholipid antibody syndrome: 6 months of observation. Auto Immun Highlights.

[6] Stafforini DM, McIntyre TM, Carter ME, Prescott SM (1987). Human plasma platelet-activating factor acetylhydrolase. Association with lipoprotein particles and role in the degradation of platelet-activating factor. J Biol Chem.

[7] McIntyre TM, Prescott SM, Stafforini DM (2009). The emerging roles of PAF acetylhydrolase. J Lipid Res.

[8] Rosenson RS, Stafforini DM (2012). Modulation of oxidative stress, inflammation, and atherosclerosis by lipoprotein-associated phospholipase A2. J Lipid Res.

[9] Garza CA, Montori VM, McConnell JP, Somers VK, Kullo IJ, Lopez-Jimenez F (2007). Association between lipoprotein-associated phospholipase A2 and cardiovascular disease: a systematic review. Mayo Clin Proc.

[10] Corson MA, Jones PH, Davidson MH (2008). Review of the evidence for the clinical utility of lipoprotein-associated phospholipase A2 as a cardiovascular risk marker. Am J Cardiol.

[11] Maiolino G, Pedon L, Cesari M, Frigo AC, Wolfert RL, Barisa M, Pagliani L, Rossitto G, Seccia TM, Zanchetta M, Rossi GP (2012). Lipoprotein-associated phospholipase A2 activity predicts cardiovascular events in high risk coronary artery disease patients. PLoS ONE.

[12] Grenn RC, Yalavarthi S, Gandhi AA, Kazzaz NM, Núñez-Álvarez C, Hernández-Ramírez D, Cabral AR, McCune WJ, Bockenstedt PL, Knight JS (2016). Endothelial progenitor dysfunction associates with a type I interferon signature in primary antiphospholipid syndrome. Ann Rheum Dis.

[13] Ambrosino P, Lupoli R, Di Minno A, Iervolino S, Peluso R, Di Minno MN (2014). Markers of cardiovascular risk in patients with antiphospholipid syndrome: a meta-analysis of literature studies. Ann Med.

[14] da Silva FF, Levy RA, de Carvalho JF (2014). Cardiovascular risk factors in the antiphospholipid syndrome. J Immunol Res.

[15] Zhang X, Xie Y, Zhou H, Xu Y, Liu J, Xie H, Yan J (2014). Involvement of TLR4 in oxidized LDL/β2GPI/anti-β2GPI-induced transformation of macrophages to foam cells. J Atheroscler Thromb.

[16] Li J, Chi Y, Liu S, Wang L, Wang R, Han X, Matsuura E, Liu Q (2014). Recombinant domain V of β2-glycoprotein I inhibits the formation of atherogenic oxLDL/β2-glycoprotein I complexes. J Clin Immunol.

[17] Pengo V, Tripodi A, Reber G, Rand JH, Ortel TL, Galli M, de Groot PG (2009). Update of the guidelines for lupus anticoagulant detection. Subcommittee on lupus anticoagulant/antiphospholipid antibody of the scientific and standardisation committee of the international society on thrombosis and haemostasis. J Thromb Haemost.

[18] Poz A, Pradella P, Azzarini G, Santarossa L, Bardin C, Zardo L, Giacomello R (2016). Lupus anticoagulant: a multicenter study for a standardized and harmonized reporting. Blood Coagul Fibrinolysis.

[19] Libby P, Bornfeldt KE, Tall AR (2016). Atherosclerosis: successes, surprises, and future challenges. Circ Res.

[20] Mahler M, Norman GL, Meroni PL, Khamashta M (2012). Autoantibodies to domain 1 of beta 2 glycoprotein 1: a promising candidate biomarker for risk management in antiphospholipid syndrome. Autoimmun Rev.

[21] Agrawal V, Jaiswal MK, Ilievski V, Beaman KD, Jilling T, Hirsch E (2014). Platelet-activating factor: a role in preterm delivery and an essential interaction with Toll-like receptor signaling in mice. Biol Reprod.

[22] O’Donoghue ML, Braunwald E, White HD, Lukas MA, Tarka E, Steg PG, Hochman JS, Bode C, Maggioni AP, Im K, Shannon JB, Davies RY, Murphy SA, Crugnale SE, Wiviott SD, Bonaca MP, Watson DF, Weaver WD, Serruys PW, Cannon CP, Steen DL, SOLID-TIMI 52 Investigators (2014). Effect of darapladib on major coronary events after an acute coronary syndrome: the SOLID-TIMI 52 randomized clinical trial. JAMA.

[23] Wallentin L, Held C, Armstrong PW, Cannon CP, Davies RY, Granger CB, Hagström E, Harrington RA, Hochman JS, Koenig W, Krug-Gourley S, Mohler ER, Siegbahn A, Tarka E, Steg PG, Stewart RA, Weiss R, Östlund O, White HD, Investigators STABILITY (2016). Lipoprotein-associated phospholipase A2 activity is a marker of risk but not a useful target for treatment in patients with stable coronary heart disease. J Am Heart Assoc.

[24] Marathe GK, Pandit C, Lakshmikanth CL, Chaithra VH, Jacob SP, D’Souza CJ (2014). To hydrolyze or not to hydrolyze: the dilemma of platelet-activating factor acetylhydrolase. J Lipid Res.

[25] Bećarević M, Ignjatović S (2016). Proinflammatory proteins in female and male patients with primary antiphospholipid syndrome: preliminary data. Clin Rheumatol.

[26] Amengual O, Forastiero R, Sugiura-Ogasawara M, Otomo K, Oku K, Favas C, Delgado Alves J, Žigon P, Ambrožič A, Tomšič M, Ruiz-Arruza I, Ruiz-Irastorza G, Bertolaccini ML, Norman GL, Shums Z, Arai J, Murashima A, Tebo AE, Gerosa M, Meroni PL, Rodriguez-Pintó I, Cervera R, Swadzba J, Musial J, Atsumi T (2016). Evaluation of phosphatidylserine-dependent antiprothrombin antibody testing for the diagnosis of antiphospholipid syndrome: results of an international multicentre study. Lupus.

[27] Maiolino G, Bisogni V, Rossitto G, Rossi GP (2015). Lipoprotein-associated phospholipase A2 prognostic role in atherosclerotic complications. World J Cardiol.

